# Comparison of outcomes between off-pump and on-pump coronary artery bypass graft surgery using collaborative targeted maximum likelihood estimation

**DOI:** 10.1038/s41598-024-61846-1

**Published:** 2024-05-18

**Authors:** Hossein Ali Adineh, Kaveh Hoseini, Iraj Zareban, Arash Jalali, Maryam Nazemipour, Mohammad Ali Mansournia

**Affiliations:** 1 Department of Epidemiology and Biostatistics, School of Public Health, Tehran University of Medical Sciences, Tehran, Iran; 2 Tehran Heart Center, Cardiovascular Diseases Research Institute, Tehran University of Medical Sciences, Tehran, Iran; 3 Cardiac Primary Prevention Research Center, Cardiovascular Diseases Research Institute, Tehran University of Medical Sciences, Tehran, Iran; 4 Health Promotion Research Center, Zahedan University of Medical Sciences, Zahedan, Iran

**Keywords:** C-TMLE, Off-pump, TMLE, Risk ratio, Cardiology, Medical research, Risk factors

## Abstract

There are some discrepancies about the superiority of the off-pump coronary artery bypass grafting (CABG) surgery over the conventional cardiopulmonary bypass (on-pump). The aim of this study was estimating risk ratio of mortality in the off-pump coronary bypass compared with the on-pump using a causal model known as collaborative targeted maximum likelihood estimation (C-TMLE). The data of the Tehran Heart Cohort study from 2007 to 2020 was used. A collaborative targeted maximum likelihood estimation and targeted maximum likelihood estimation, and propensity score (PS) adjustment methods were used to estimate causal risk ratio adjusting for the minimum sufficient set of confounders, and the results were compared. Among 24,883 participants (73.6% male), 5566 patients died during an average of 8.2 years of follow-up. The risk ratio estimates (95% confidence intervals) by unadjusted log-binomial regression model, PS adjustment, TMLE, and C-TMLE methods were 0.86 (0.78–0.95), 0.88 (0.80–0.97), 0.88 (0.80–0.97), and 0.87(0.85–0.89), respectively. This study provides evidence for a protective effect of off-pump surgery on mortality risk for up to 8 years in diabetic and non-diabetic patients.

## Introduction

Atherosclerosis is a progressive chronic inflammatory disease of the vessels, which causes the thickness of the arterial wall to increase^1^. The myeloid cells destabilize the lipid-rich plaques in the artery wall and cause myocardial infarction^2^.

Coronary artery bypass grafting (CABG) was introduced in 1968 and rapidly accepted as the standard treatment for patients with atherosclerosis. Percutaneous coronary intervention (PCI) was introduced in 1977 to the medical community as well. Applying these treatments has reduced morbidity and mortality^3,4^.

CABG has been generally performed with the use of cardiopulmonary bypass (on-pump) machine and operating on a beating heart (off-pump) techniques^5^. Studies of these two types of CABG and a comparison of their effect on mortality and complications such as myocardial infarction, and stroke revealed no short-term differences between the approaches, while some other studies showed a more promising effect in long-term outcomes for the off-pump technique^2,4^. In contrast, some studies advocate the no advantages of off-pump CABG for 10-year death or revascularization outcome and manifest lower time to composite endpoint in the off-pump group than in the on-pump group^6,7^. There are many controversies and debates about the risks and benefits of CABG with cardiopulmonary bypass^8^. Moreover, the effect of CABG with and without cardiopulmonary bypass on mortality among diabetes and non-diabetes patients need more research^9^.

Confounding can be a major reason for this ambiguity about off-pump consequences. Conventional methods rely on correct specification of outcome model and can lead to biased and confounded results. There are numerous approaches for addressing this problem e.g., propensity score (PS) methods, g-formula or double-robust methods such as targeted maximum likelihood estimation (TMLE)^10–31^. PS methods and g-formula rely on exposure and outcome modelling, respectively, and TMLE uses both of these models^32^. Failure to specify the correct model in PS methods and the bias-variance trade-off issue in TMLE leads to biased estimates for the parameter of interest and high variance when analysing a large number of variables, respectively^33,34^. Since the estimates from sparse data, regression of exposure given confounders is close to zero or one within one or more strata of covariates, can be biased, double robust estimators are not immune to light violations of the positivity assumption in sparse data. The collaborative targeted maximum-likelihood estimation (C-TMLE) addresses biased estimates from sparse data by applying the nuisance parameter estimation and decreasing mean square error (MSE) in the parameter estimate .^35^ C-TMLE method does not require that either the estimate of exposure or outcome be consistent, rather it focuses on the reduction of the distance between the estimate of exposure mechanism and its parameter and outcome estimate and its parameter, therefore this approach provides an unbiased and precise estimate (low standard error or narrower confidence interval) of the parameter^34^. Since C-TMLE produces stable estimates of borderline identifiable parameters and is super-efficient and also because of ambiguities about the effect of off-pump we aimed to use a stronger and more optimized double robust method, C-TMLE, for address a selected- least set of covariates and estimate the effect of the off-pump technique surgery on mortality.

## Methods

### Study oversight and setting

We examined the effect of off-pump CABG on death by a prospective analysis of Tehran Heart Center’s Surgery data-registry with a median follow-up of 8 years. From 2007 to 2016, patients having undergone isolated CABG were involved in the cohort study by protocol and then were followed in 4, 6, and 12 postoperative months and annually afterward. Follow-ups were done by inpatient visits and face-to-face interviews or telephone contacts for patients who could not attend clinic. The data are stored in the CABG Follow-up databank, which contains classified variables such as demographics, atherosclerosis risk factors, the status of their control, ECG and routine laboratory findings, exercise test results, major events such as acute coronary syndrome, mortality, etc. The Tehran Heart Center (THC)is a major medical treatment facility in Iran that serves both local patients and those referred from other cities. In THC, 28,945 subjects with isolated first-time CABG procedures and with at least 1 graft that has been followed between March 2007 and March 2020^36^.Of these, 4062 patients with incomplete registry data, loss to complete follow-up or CABG with valve surgery were excluded. Finally, 24,883 participants were included in the analysis.

To ensure obscurity, personal identifiers such as name, phone number, and residence address were removed from the raw data, and personal identification number was converted into a serial number. Moreover, this study was approved in terms of the study protocol by the Tehran Heart Center (THC) Review Board and the ethical committee of the Tehran University of Medical Sciences (IR.TUMS.SPH.REC.1402.004). At the time of hospitalization, all patients gave written and informed consent, which included prolonged data collection in the follow-up. Using data in the present study were according to the Helsinki Declaration.

### Definition of variables

We defined blood hypertension as systolic blood pressure ≥ 140 mm Hg or diastolic blood pressure ≥ 90 mm Hg or a history of prescribed antihypertensive medications. Additionally, we labeled diabetes mellitus (DM) as any one of fasting plasma sugar ≥ 126 mg/dl; or HbA1c ≥ 6.5%; taking oral hypoglycemic drugs; or injecting insulin; or random plasma glucose ≥ 200 mg/dl, any type of diabetes was considered as diabetes (9941, 39.9%). Ejection fraction was classified as normal (heart failure ≥ 55 in men and ≥ 60 in women), mildly reduced, and reduced (heart failure < 40%)^13^. Each patient with one of the following disorders was involved in the dyslipidemia group; total cholesterol level ≥ 240 mg/dl; LDL-C level of more than 160 mg/dl; triglyceride serum ≥ 200 mg/dl; and HDL-C of less than 40 mg/dl and less than 50 mg/dl in men and women, respectively; or a history of taking lipid medications based on the National Cholesterol Education Program (NCEP) Adult Treatment Plan (ATP) III^37,38^. According to the patient's self-response, we classified cigarette smoking status as never smoker and before or current smoker. Moreover, patients were labeled as opium-addicted if they reported current consumption of opium, either inhalation or oral or drinking (with water or tea). The Body Mass Index (BMI) was calculated as the individual's weight in kilograms divided by the square of the height in meters. COPD was diagnosed as incompletely reversible airflow obstruction which was confirmed by spirometry with a ratio of post-bronchodilator forced expiratory volume in one second to forced vital capacity (FEV1/FVC), 0.7, in the lack of bronchiectasis or tuberculosis to otherwise. Additionally, we consider a family history of CAD as acute myocardial infarction or recorded CAD, diagnosed by either computed tomography or invasive coronary angiography, in first-degree relatives.

### Exposure

Patients with CABG on the beating heart without cardiopulmonary bypass, and off-pump coronary artery bypass (OPCAB), were defined as the exposure group. In contrast, performed CABG on patients using the on-pump technique, with cardiopulmonary bypass^39^, was classified in the unexposed group.

### Outcome

Our outcome was all deaths following surgery, evaluated as both dichotomous (Death = 0 and Death = 1) and time-to-event endpoints, from the date of surgery up to the last follow-up date. In addition, subjects with any information about the outcome were considered as right censored.

### Causal diagram

**Figure 1:**
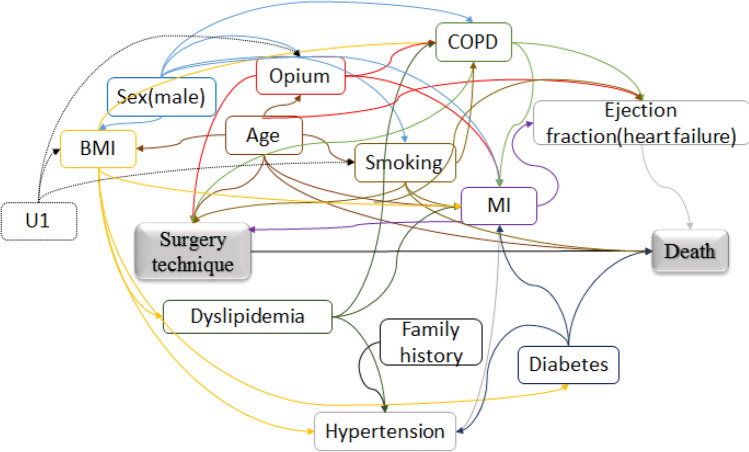
Causal directed acyclic graph for the effect of the surgery technique on death.

As shown in Fig. 1, the technique of surgery (Off-pump/on-pump) and death were considered as the main exposure and outcome of the study, respectively. Then the causal directed acyclic graph (cDAG) or causal diagram illustrating the causal relationships between exposure, outcome, and covariates, used for selecting a minimally sufficient set of confounders^40–48^. The presence of an arrow from a particular variable to another variable indicates that there is a causal direct effect. The variables age, COPD, pre or current myocardial infarction, smoking, and consumption of opium were considered as the minimum sufficient set which was included in the adjustment model. Smoking and opium are associated with cardiac function decline. For patients with ventricular dysfunction, off-pump CABG has better outcomes^49^. The unmeasured variables in our study were indicated as U and directed by a dotted arrow (U1 is lifestyle). Additionally, to better contrast variables were depicted by different colors (Fig. 1).

### Analysis

Normally distributed and non-Normally distributed variables were presented as mean (SD) and median (IQR), respectively. Normality was assessed by the Kolmogorov–Smirnov test and Q-Q plot^50^. Moreover, log-binomial regression model was used for estimating unadjusted/adjusted risk ratio. Independent Student's t-test was performed for comparison of continuous variables with Normal distribution and Mann–Whitney U-test was performed for not Normally distributed. Additionally, the Pearson χ2 test was conducted for categorical variables. Markov Chain Monte Carlo-based method was used to impute the missing data of BMI (80 missing values, 0.30%), smoking status (49 missing values, 0.19%), MI (92 missing values, 0.37%), COPD (80 missing values, 0.30%), and family history (31 missing values, 0.12%). All variables in Table 1 were involved in the imputation process.

**Table 1:** Descriptive characteristics of the patients (n = 24,803) in Tehran Heart Cohort study based on surgery technique.

Variable /groups	Off-pump	On-pump	*P*-value
	N = 1881	N = 22,922	
Death status			
Dead	367(19.51)	5176(22.58)	0.0021
Alive	1514(80.49)	17,746(77.42)	
Sex			
Female	513(27.27)	6033(26.32)	0.36
Male	1368(72.73)	16,889(73.68)	
BMI			
≤ 30	1382(73.47)	17,411(75.96)	0.015
> 30	499(26.53)	5511(24.04)	
Smoking			
Never	1209(64.86)	14,540(63.52)	0.24
Current/before	655(35.14)	8350(36.48)	
Opium consumption			
Never	1570(84.27)	19,368(84.65)	0.66
Current /before	293(15.73)	3511(15.35)	
COPD			
Yes	84(4.53)	836(3.66)	0.056
Dyslipidemia			
Yes	1164(61.88)	14,933(65.16)	0.0041
Hypertension			0.083
Yes	1088(57.96)	12,807(55.9)	
MI $			
Yes	624(33.84)	9802(82.87)	< 0.0001
Family history #			
Yes	603(32.13)	9240(40.36)	< 0.0001
Ejection fraction			
Normal	718(38.17)	10,132(44.2)	
Mildly reduced	535(28.44)	6518(28.44)	< 0.0001
Reduced	628(33.39)	6272(27.36)	
Age; mean (SD)	65(10.1)	65(10.0)	0.8
Follow-up time; median (IQR)	6.3(5.0,7.3)	8.3(5.8,11.2)	< 0.0001

No. (%) has been reported for description of the variables, unless otherwise stated. $ Myocardial Infarction # History of cardiovascular disease, MI, and stroke in first-degree relatives.

Pearson χ2 test, Independent Student's t-test, and Mann–Whitney U-test.

In the first step, TMLE in general form (event = 0 and event = 1) models the outcome (death) based on exposure (technique of surgery) and confounders (age, COPD, pre or current myocardial infarction, smoking, and consumption of opium) using super learner (initial estimate). Then, using a regression model of exposure on confounders (super leaner), PS is estimated and a clever covariate, known as H, is generated (step 2). In this model, the technique of surgery (exposure) is regressed on age, COPD, pre or current myocardial infarction, smoking, and consumption of opium. The predicted probability of the fitted model is PS. In the last step, the outcome model is updated (step 1) by refitting step 1 and including H in model; in the updated model, initial estimate is considered as an offset. Finally, risk ratio is estimated using standardized mean in the exposed and un-exposed groups based on the updated outcome model.

C-TMLE is extension of TMLE which attempts to avoid increasing variance by sequentially updating outcome regressions based on PS estimates that incorporate an increasing number of confounders^51^. The general C-TMLE procedure is based on the following steps;

In the first step, an estimate of outcome model (${\widehat{Q}}^{0}$) on exposure (technique of surgery) and confounders (all confounders) is obtained using super learner.

In the second step, a sequence of regression model of exposure ($\widehat{g}$) is generated, so that the confounders are included in the exposure model in a stepwise manner based on the cross-validated penalized likelihood of the outcome model .(${\widehat{g}}^{1}$ : age , ${\widehat{g}}^{2}:age and COPD, {\widehat{g}}^{3}:age,COPD,$ and pre or current myocardial infarction, ${\widehat{g}}^{4}$:$age, COPD,$ pre or current myocardial infarction, and smoking,${\widehat{g}}^{5}: age,COPD,$ pre or current myocardial infarction, smoking, and consumption of opium.). The first clever covariate is constructed by adding a confounder, the one which maximizes the penalized likelihood of the outcome model, to the exposure model.(${\widehat{g}}^{1}$ : age).

In the third step, the outcome regression model, fitted in the first step, is updated based on the first clever covariate, with initial estimate of step 1 as offset. Next, exposure model is updated by including an additional confounder (${\widehat{g}}^{2}:age and COPD$). This process continues until the log-likelihood of the outcome model does not increase (until ${\widehat{g}}^{5}$ in our analysis). In the Final step, risk ratio is estimated using standardized mean in the exposed and un-exposed groups based on the last updated outcome model. identifying TMLE (${\widehat{Q}}^{*}$), the candidate TMLE in the sequence that minimizes the cross-validated risk and finally estimating effect measure by using outcome mean in exposure and un-exposure groups^34^.

For comparison, we estimated the PS for each patient using multivariable logistic regression model. To compare models, we calculated Akaike information criterion (AIC). A minimally sufficient set of confounders based on Fig. 1 was included as predictors and the variable surgery technique (off-pump/on-pump) was involved as the response variable. Using the PS and exposure variable in the log-binomial model, we estimated the PS-adjusted effect. Additionally, adjusted Cox proportional hazards model was performed. Standard errors for TMLE and C-TMLE were estimated using influence function, and all effect estimates were reported as risk ratio with 95% CIs^52–57^. All analyses were repeated in diabetes and non-diabetes subgroups. Statistical analysis was performed using R, version 3.5.0 (R Foundation for Statistical Computing, Vienna, Austria).

## Results

During the follow-up, with the median (Q1, Q3) of 8 (5.6, 11.1) years, 24,883 patients 18,318 male and 6565 female underwent isolated coronary artery surgery and were included in the Tehran Heart Center’s CABG data registry. The mean (SD) age of the study participants was 65 (10.0) years, ranged from 19 to 95 years (more than 96% of patients were older than 30 years). A total of 1881 patients underwent off-pump surgery 367 (19.5%), and from 22,922 patients with on-pump, 5176 (22.6%) died during the study follow-up. The on-pump group had a longer follow-up time (8.3 (5.8, 11.2) and 6.3(5.0, 7.3), respectively). Moreover, participants with off-pumps were more likely to have a BMI higher than 30 kg/m^2^ (26.5% vs. 24.0%). On-pump coronary artery bypass graft surgery was associated with having a myocardial infarction, family history of CVD, and dyslipidemia. In contrast, participants who experienced a lower ejection fraction were more likely to have off-pump surgery (Table 1).

Moreover, we assessed the association between study variables and surgery technique in both diabetic and non-diabetic patients. As shown in Table 2, both diabetic and non-diabetic participants who underwent on-pump CABG were more likely to die than the off-pump group. In addition, the high body mass index was associated with taking off-pump surgery in non-diabetic patients. Moreover, Table 2 indicates that patients with diabetes who have hypertension had an increased probability of undergoing off-pump CABG.

**Table 2:** Association between study variables and technique of surgery in diabetes subgroups.

Variables /Groups	Diabetics		*P*-value	Non-diabetics		*P*-value
	Off-pump n = 779	On-pump n = 9162		Off-pump	On-pump	
				n = 1102	n = 13,760	
Death status						
Dead	189(24.3)	2519(27.5)	0.052	178(16.1)	2657(19.3)	0.01
Alive	590(75.7)	6643(72.5)		924(83.9)	11,103(80.7)	
Sex						
Female	428(37.42)	289(37.1)	0.86	224(20.33)	2605(18.93)	0.25
Male	734(67.58)	490(63.0)		878(79.67)	11,155(81.07)	
BMI						
≤ 30	557(71.5)	6751(73.68)	0.18	825(74.86)	10,660(77.47)	0.046
> 30	222(28.5)	2.411(26.32)		277(25.14)	3100(22.53)	
Smoking						
Never	546(71.19)	6482(70.86)	0.85	663(60.44)	8058(58.63)	0.24
Current/before	221(28.81)	2665(29.14)		434(39.56)	5685(41.37)	
Opium consumption						
Never	678(88.4)	7977(87.26)	0.36	892(81.4)	11,391(82.9)	0.19
Current /before	89(11.6)	1165(12.74)		204(18.6)	2346(17.1)	
COPD						
Yes	37(4.84)	333(3.65)	0.096	47(4.3)	503(3.7)	0.27
Dyslipidemia						
Yes	565(72.5)	6908(75.4)	0.071	599(54.4)	8025(58.3)	0.01
Hypertension						
Yes	528(68.1)	5905(64.5)	0.043	560(50.8)	6902(50.1)	0.68
MI^$^						
Yes	247(32.5)	3799(41.6)	< 0.0001	377(34.8)	6003(43.71)	< 0.0001
Family history #						
Yes	241(30.9)	3798(41.5)	< 0.0001	362(32.9)	5442(39.6)	< 0.0001
Ejection fraction						
Normal	294(37.74)	4245(46.33)		424(38.48)	5887(42.78)	
Mildly reduced	195(25.0)	2221(24.24)	< 0.0001	340(30.85)	4297(31.23)	0.002
Reduced	290(37.2)	2696(29.43)		338(30.67)	3576(25.99)	
Age; mean (SD)	66(9.14)	66 (9.14)	0.29	65(10.7)	65(10.4)	0.26
Follow-up time; median (IQR)	6.2(4.9,7.3)	8.1(5.4,10.6)	< 0.0001	6.3(5.2,7.3)	8.4(6.0,11.0)	< 0.0001

No. (%) has been reported for description of the variables, unless otherwise stated.

$Myocardial Infarction.

#History of cardiovascular disease, MI, and stroke in first-degree relatives.

Pearson χ2 test, Independent Student's t-test, and Mann–Whitney U-test.

**Figure 2:**
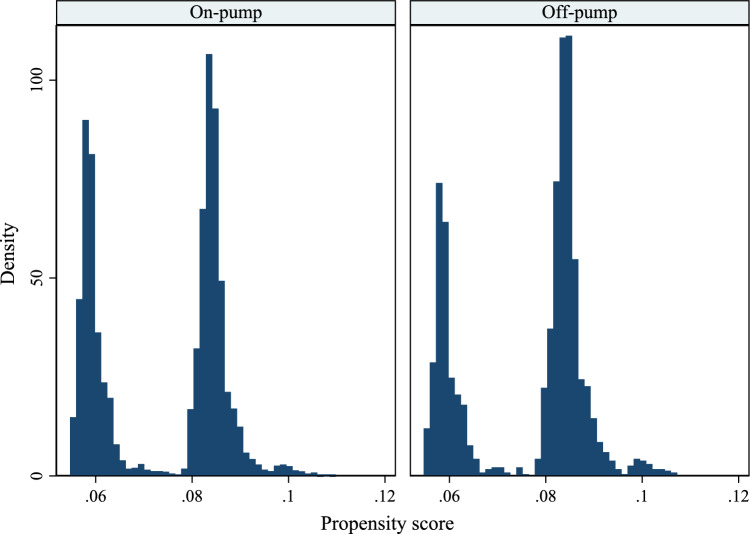
Histogram distribution of propensity score by surgery technique.

To assess the potential violation of the positivity assumption, the distribution of PS for both on-pump and off-pump groups has been depicted in Fig. 2. The median (IQR) for both groups are 0.081(0.059, 0.084) and 0.082 (0.060, 0.084), respectively. The minimum value (0.054) and maximum value (0.1) were almost equal. In general, both distributions are very similar so that PS values were mostly lower than 0.1 and bimodal nearly in 0.06 and 0.08.

The effect estimates of surgery technique on the risk of mortality along with 95% CIs have been estimated by four statistical methods, presented in Table 3: unadjusted, PS-adjusted, TMLE and C-TMLE, for subgroups of diabetes and non-diabetes patients as well as Total sample.

**Table 3:** Sub-group analysis: risk ratio estimates (95% CIs) between surgery technique and mortality in the Tehran Heart study (THS).

Method	RR	95% CI	AIC	SE	P-value
Diabetic patients (n = 9982)					
Unadjusted	0.88	(0.77–1.00)	11,640	0.057	0.056
PS-adjusted	0.89	(0.78–1.02)	11,522	0.059	0.110
TMLE	0.88	(0.77–1.00)	11,228	0.064	0.051
C-TMLE	0.87	(0.84–0.90)	11,238	0.015	< 0.001
Adjusted COX PH	1.31^†^	(1.10–1.55)	40,326	0.11	0.002
Non-diabetic patients (n = 14,901)					
Unadjusted	0.83	(0.72–0.96)	14,480	0.059	0.010
PS-adjusted	0.87	(0.76–1.00)	14,264	0.056	0.057
TMLE	0.88	(0.73–1.01)	13,519	0.067	0.078
C-TMLE	0.86	(0.83–0.88)	13,528	0.011	< 0.001
Adjusted COX PH	1.21^†^	(1.02–1.43)	44,600	0.10	0.024
Total (n = 24,883)					
Unadjusted	0.86	(0.78–0.95)	26,346	0.041	0.003
PS-adjusted	0.88	(0.80–0.97)	26,062	0.043	0.0091
TMLE	0.88	(0.81–0.97)	25,036	0.047	0.010
C-TMLE	0.87	(0.85–0.89)	25,022	0.009	< 0.001
Adjusted COX PH	1.24^†^	(1.10–1.40)	91,956	0.075	< 0.001

RR (risk ratio), AIC (Akaike information criterion), PS-adjusted (Propensity score full adjustment), Cox proportional hazards model, TMLE (Targeted maximum likelihood estimation), C-TMLE (Collaborative Targeted Maximum Likelihood Estimation). †Hazard ratio.

The Cox proportional hazards model shows a higher hazard of mortality for the off-pump technique, with HR = 1.31, 95% CI 1.10–1.55 in diabetes patients, HR = 1.21, 95% CI 1.02–1.43 in non-diabetes patients, and HR = 1.24, 95% CI 1.10–1.40 in the total sample. Proportional hazards assumptions were confirmed using scaled Schoenfeld residuals (The *p*-values were 0.13, 0.73, and 0.26 in the total, diabetes, and non-diabetes, respectively).

Considering 95% CI, PS-adjusted and TMLE revealed evidence for weak protective effect of off-pump CABG, consistently in diabetic and non-diabetic groups as well Total sample. However, this evidence of effect was stronger based on C-TMLE. As shown in Table 3, double robust methods (TMLE and C-TMLE) exhibit a better fit for the data (lower AIC). Additionally, it is important to note that the most precise effect estimates were provided by C-TMLE.

## Discussion

In this large prospective registry-based cohort study, 24,883 patients (1881 off-pump and 22,922 on-pumps) underwent CABG surgery and were followed up for an average of 8.2 years. According to the C-TMLE, there was an evidence of protective effect of the off-pump surgery on mortality: in the Total sample risk was decreased at least about 10%, consistently in diabetic and non-diabetic patients. This evidence was less clear based on PS adjusted and TMLE analyses.

Conventional analysis revealed that off-pump surgery was associated with lower mortality and higher body mass index. These findings were in agreement with some previous researches claiming off-pump CABG is associated with higher survival, including the Oxford database study^58^ and the Emory Healthcare Hospitals data analysis^59^. Our study demonstrated that most patients who had previous or current myocardial infarction or a history of CVD were more likely to have on-pump CABG. It should be noted that the choice between off-pump and on-pump seems to be surgeon-dependent^60^. Our findings were in the same direction with the study that reported off-pump CABG as a superior technique in reducing mortality over the on-pump in patients with diabetes^61^. Totally, the unadjusted analysis suggested a protective effect which is similar to the findings of previous observational studies^62^. We assumed that these results may be affected by several potential confounders such as age and smoking that were not controlled, correctly. To overcome the bias induced, the adjusted risk ratio was calculated using PS-adjusted and TMLE, both of which indicated a protective effect for off-pump CABG, Also, C-TMLE provided a stronger evidence regarding a protective effect of off-pump vs on-pump surgery. C-TMLE can address potential biased estimates from light sparse data by applying the nuisance parameter estimation and also, decrease mean square error (MSE) in parameter estimate. Accordingly, the estimated risk ratio by C-TMLE is more confident^8,35,63^.

On the contrary, the Cox proportional hazard model shows a higher hazard ratio of mortality for off-pump procedures. This could be due to the fact that the hazard ratio of mortality varies over time. By definition, hazard ratio is conditional on survival and so is inherently subject to selection bias if there is an unmeasured risk factor of mortality, even independent of the exposure at the start of follow-up^64^.

Substantial strengths of our study were applying a precise double-robust method for efficient adjustment of confounders, low bias estimation, large sample size, and low proportion of missing covariates (< 0. 1%). Our study has the following limitations. First, time-to-death data were not assessed. Second, there was a possibility of risk fluctuation as there were some time-dependent covariates^65,66^. Third, our minimal sufficient confounders were self-reported; specifically opium consumption and cigarette smoking, so there was a possibility of residual confounding due to measurement error^67,68^. Fourth, the confounding effect of drugs was missed. Last, limitations to the generalizability of the results because of the quality of training for pump staff, type of hospital (governmental, private, and teaching) and availability and cost of equipment required for on-pump surgery may be different among countries.

## Comments

This study provides evidence for a protective effect of off-pump on mortality risk for up to 8 years. Further research on survival time using C-TMLE is needed.

## Data Availability

The data used and analysed during the present study are accessible from the corresponding author in STATA and SPSS format if required.
